# Evolution Law of Structural Form and Heat Transfer Performance of Thermal Insulation System

**DOI:** 10.3390/ma16186341

**Published:** 2023-09-21

**Authors:** Shuang-Xi Zhou, Jian-Xin Li, Shu-Feng Bao, Yang Ding, Yong-Qi Wei, An-Ming She, Zhen-Zhen Guo, Jing-Liang Dong

**Affiliations:** 1 School of Civil and Engineering Management, Guangzhou Maritime University, Guangzhou 510725, China; 2 Department of Civil Engineering, Hangzhou City University, Hangzhou 310015, China; 3 School of Materials Science and Engineering, Tongji University, Shanghai 201804, China; 4 School of Civil Engineering and Architecture, East China Jiao Tong University, Nanchang 330013, China

**Keywords:** insulation system, thermal conductivity experiment, numerical calculation of thermal conductivity, structural form, COMSOL finite element simulation

## Abstract

Building thermal insulation and energy conservation have become urgent problems in the field of civil engineering because they are important for achieving the goal of carbon neutralization. Thermal conductivity is an important index for evaluating the thermal insulation of materials. To study the influence of different porosity levels on the thermal conductivity of materials, this paper established a random distribution model using MATLAB and conducted a comparative analysis using COMSOL finite element software and classical theoretical numerical calculation formulas. The thermal conductivity of composite materials was determined based on a theoretical calculation formula and COMSOL software simulations, and the theoretical calculation results and simulation results were compared with the measured thermal conductivity of the composites. Furthermore, the influence of the width of the gaps between the materials on the heat transfer process was simulated in the fabricated roof structure. The results showed the following: (1) The thermal conductivity values calculated using the Zimmerman model were quite different from those calculated using the Campbell-Allen model and those calculated using the COMSOL software; (2) The thermal conductivity values calculated using the theoretical calculation formula were lower than the measured data, and the maximum relative error was more than 29%. The COMSOL simulation results were in good agreement with the measured data, and the relative error was less than 5%; (3) When the gap width was less than 60 mm, it increased linearly with the heat transfer coefficient. The heat transfer coefficient increased slowly when the gap width was greater than 60 mm. This was mainly due to the thermal bridge effect inside the insulation system. Based on these research results, a thermal insulation system was prepared in a factory.

## 1. Introduction

Following the proposal of the green dual carbon goal, civil engineering projects such as buildings, bridges, and tunnels have been actively conducting research on green energy conservation and emission reduction [1,2,3,4,5,6]. Building energy conservation is a fundamental national policy advocated by China’s energy law [7,8]. Currently, building energy consumption in China accounts for approximately 30% of the total terminal energy consumption of the entire country, and the energy consumption per unit area is two to five times higher than that of developed countries [9,10]. The energy consumption of building envelopes and roofs accounts for more than 60% of building energy consumption [11,12]. Therefore, research on roof insulation materials plays a crucial role in the development of green buildings.

The Chinese rigid polyurethane foam (RPUF) industry began to develop in the late 1950s [13]. RPUF has excellent thermal insulation performance and does not exhibit significant deformation under small loads, and this has led to its rapid development in recent years [14]. During the same period, foam concrete (FC) technology was also popularized in China [15,16]. FC has better compressive strength and aging resistance than traditional chemical thermal insulation materials [17], making it an ideal replacement for traditional thermal insulation materials [18]. In recent years, with the growing popularity of vacuum insulation panels (VIPs), the building insulation industry has experienced rapid development [19,20]. The thermal conductivity of VIPs developed by Chinese enterprises can reach below 0.006 W/(mK), and their thermal insulation performance is 6–10 times that of traditional thermal insulation materials [21]. Additionally, the service life of VIPs can reach 60 years, and they are biodegradable and thus suitable for secondary recycling [22]. The entire production process meets the requirements of green environmental protection goals, and they provide excellent thermal insulation performance [23]. VIPs are a commonly used insulation material [24].

This paper focuses on three thermal insulation materials, namely RPUF, FC, and VIPs, and analyzes their energy-saving performance in various insulation systems. The paper begins by introducing the current research status of these three materials. This is followed by an introduction to common thermal conductivity calculation theories and the COMSOL (https://www.comsol.com/) finite element simulation calculation method. The two methods are then compared through tests and calculations. Finally, the paper discusses the impact of different structural forms on the energy-saving performance of thermal insulation systems and determines the final industrial product.

## 2. Overview of Thermal Insulation Materials

### 2.1. Rigid Polyurethane Foam

The excellent thermal insulation properties of RPUF are due to the closed pores formed during foaming, which are filled with freon gas. This gas has a much smaller thermal conductivity than air [25,26]. The size and distribution of these pores also play crucial roles in the material’s thermal insulation performance [27]. In general, RPUF exhibits the following basic characteristics [28,29]:

(1) Regularly arranged closed pores. This is the main form of RPUF, and the pore size is approximately 50 nm. The existence of these closed pores allows foamed plastics to exhibit good thermal insulation performance. However, the cell wall can absorb the foaming agent gas inside the cell, which can affect the thermal conductivity of the material.
(2) Irregular-shaped vesicles with no obvious hybrid structure. This structure often occurs due to local polymerization and imperfect foaming processes, resulting in dead zones.

### 2.2. Foam Concrete

FC is produced by adding a foam agent to slurry [30,31]. The slurry consists of cement, aggregate, admixtures, and water [32]. Lightweight microporous concrete is formed by mixing, pouring, and curing [33]. The main properties of foamed concrete typically include the following five points [34,35]:

(1) Light weight: foamed concrete has a low density which typically ranges from 300 kg/m^3^ to 1200 kg/m^3^.
(2) Good insulation performance: foamed concrete contains a large number of closed, uniform, and fine circular pores which contribute to its excellent insulation performance.
(3) Excellent sound insulation performance: as a porous material, foamed concrete contains a large number of closed pores, which leads to superior sound insulation performance.
(4) Non-flammable and high-temperature resistant: Cement is the main raw material of foamed concrete and is classified as a class a fireproof material.
(5) Environmental benefits: foamed concrete can be mixed with industrial waste during the production process, enabling the effective use of waste resources and contributing to environmental protection efforts.

### 2.3. Vacuum Insulation Panels

VIPs are a new and efficient thermal insulation material based on the principle of vacuum thermal insulation [36]. They have been developed and studied in recent years, and they achieve thermal insulation and heat conduction by maximizing the vacuum in the board and filling it with core materials, which results in ideal thermal insulation effects for energy-saving purposes [37]. Compared with RPUF and FC, VIPs have a much lower thermal conductivity, ranging from 0.003 to 0.006 W/(mK). However, it should be noted that VIPs have a low water vapor permeability coefficient, and precautions need to be taken during construction to prevent vacuum damage [38]. If the vacuum degree is damaged, the core material may fall out. Once the vacuum degree is completely destroyed, the thermal conductivity of VIPs increases to 0.018–0.02 W/(mK).

In order to optimize the performance of VIP components during production, special attention should be given to the following three aspects [39,40]: (1) Core material: The performance of a VIP depends heavily on its core material. Therefore, it is essential to select the appropriate core material and optimize its properties to achieve the desired thermal insulation performance; (2) Gas barrier structure: The gas barrier structure of a VIP plays a crucial role in maintaining the vacuum degree. The gas barrier structure should be designed to minimize gas permeability and prevent vacuum loss, thus ensuring the long-term performance of the VIP; (3) Vacuum: The vacuum degree is closely related to the thermal performance of a VIP. Different core materials require different vacuum pressures to achieve optimal performance. To ensure a good thermal insulation effect, the vacuum pressure in the plate needs to be maintained between 1~100 Pa.

### 2.4. Materials and Test Method

**Figure 1:**
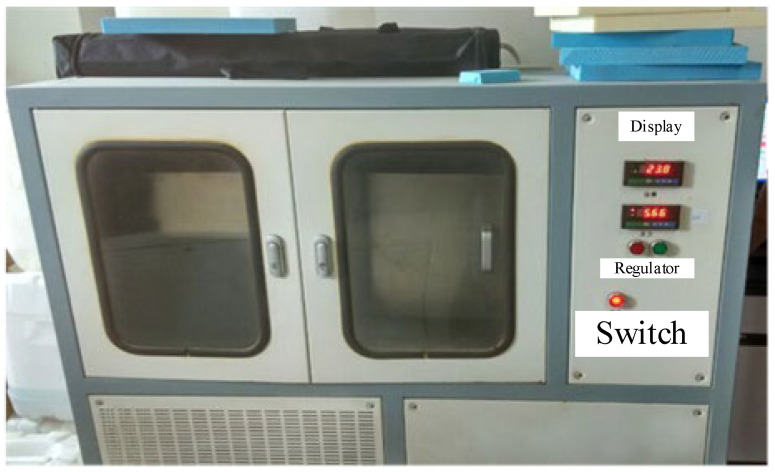
Thermal conductivity tester.

Thermal conductivity can be measured using either steady-state or dynamic methods [41]. We chose the steady-state method to test the thermal conductivity of the thermal insulation materials. The thermal conductivity measurements were carried out using a single-plate thermal conductivity tester (instrument model IMDRY300-II, produced by Tianjin Yingbei Technology Development Co., Ltd.). The thermal conductivity measurements were carried out in accordance with the national standard (GB/T 13475-2008 “Determination of Steady State Heat Transfer Properties of Thermal Insulation—Calibration and Protective Hot Box Method”), and the state was adjusted for 24 h in a temperature and humidity environment of 23 °C and 50% RH. The cold and hot end faces were put in close contact with the specimen, the pressure range was adjusted, ensuring that it did not exceed 5 kN. Before testing, the hot end temperature was set to 35 °C and the cold end temperature was set to 15 °C. First, a heat source was used to heat the sample so that the temperature inside the sample would change from a high temperature to a low temperature, as shown in Figure 1.

The temperature of the cold plate and the hot plate were set to 15 °C and 35 °C, respectively. Once a stable temperature distribution was established within the sample, the thermal conductivity tester used this temperature distribution to calculate the thermal conductivity as follows [42]:

(1) $$\frac{∆Q}{∆t}=\lambda A\frac{T_{1}-T_{2}}{h}$$

where ∆Q is heat (J), ∆t is the time difference (min), λ is the thermal conductivity (W/mk), *A* is the material contact area (m^2^), *T*_1_ is the hot plate temperature (K), *T*_2_ is the cold plate temperature (K), and *h* is the sample thickness (mm).

The raw materials used in this study included PURF produced by Nanjing Kaikai Polyurethane Co., Ltd. (Nanjing, China), FC produced by Beijing Zhongke New Building Foam Concrete Co., Ltd. (Beijing, China), and thermal insulation mortar produced by Nanjing Jinyang Energy Saving Building Materials Co., Ltd. (Nanjing, China). The manufacturer-provided values for thermal conductivity, specific heat capacity, and density are presented in Table 1.

**Figure 2:**
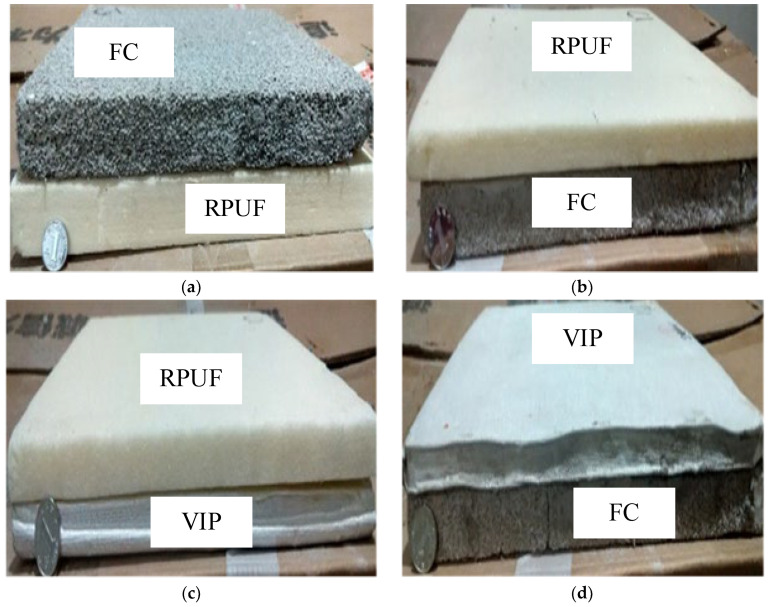
Composite material scheme. (**a**) Scheme 1; (**b**) Scheme 2; (**c**) Scheme 3; (**d**) Scheme 4. Note: Scheme 1: 300 mm × 300 mm × 25 mm FC + 300 mm × 300 mm × 2 mm mortar + 300 mm × 300 mm × 30 mm RPUF; Scheme 2: 300 mm × 300 mm × 25 mm FC + 300 mm × 300 mm × 2 mm mortar + 300 mm × 300 mm × 15 mm RPUF; Scheme 3: 300 mm × 300 mm × 15 mm RPUF + 300 mm × 300 mm × 2 mm mortar + 300 mm × 300 mm × 15 mm VIP; Scheme 4: 300 mm × 300 mm × 25 mm FC + 300 mm × 300 mm × 2 mm mortar + 300 mm × 300 mm × 15 mm VIP.

The composite insulation materials were assembled as described above, and as is shown Figure 2.

The measured data were obtained using the thermal conductivity test method and the above scheme. The data are shown in Table 2.

## 3. Numerical Simulation

### 3.1. Relationship between Porosity and Thermal Conductivity

According to Ohm’s law, the Campbell-Allen model is derived as follows [43]:

(2) $$k=k_{s}\left(2M-M^{2}\right)+\frac{k_{s}k_{a}{\left(1-M\right)}^{2}}{k_{a}M+k_{s}\left(1-M\right)}\;M=1-{\left(1-p\right)}^{1/3}$$

Zimmerman proposed a continuous medium model for calculating thermal conductivity using mean field theory, as shown in Equation (3) [44]. This model takes into account not only the porosity of FC, but also the shape of the pores.

(3) $$\frac{1}{k}=\frac{p}{k_{s}}+\frac{1-p}{k_{a}}$$

where *p* is porosity, *k* is the thermal conductivity of the material (W/mk), *k_s_* is the thermal conductivity of the solid (W/mk), and *k_a_* is the thermal conductivity of the air (W/mk).

### 3.2. Calculation of Thermal Conductivity of Composite Materials 

The thermal conductivity of a composite insulation material is the parallel connection of resistance, and it is obtained using the theoretical calculation formula (Formula (4)) [45]:

(4) $$\lambda =\frac{1}{\frac{w_{1}}{\lambda_{1}}+\frac{w_{2}}{\lambda_{2}}+\ldots \frac{w_{n}}{\lambda_{n}}}$$

where *λ*_1_, *λ*_2_, …, *λ_n_* represent the thermal conductivity of each basic material (W/mk) and *w*_1_, *w*_2_, …, *w_n_* represent the thickness as a percentage of the total thickness (mm).

### 3.3. Finite Element Software Simulation

COMSOL Multiphysics is a finite element-based simulation tool that solves partial differential equations to simulate real physical phenomena [46]. It has been widely used in various fields, including heat conduction [47]. In this paper, the solid heat conduction was simulated using the powerful finite element function of COMSOL. The formula for this simulation is as follows [48]:

(5) $$\rho C_{p}\nabla T+\nabla \left(-\lambda \nabla T\right)=Q$$

where ρ is the material density (kg/m^3^), $C_{p}$ is the specific heat capacity (J/kgK), ∇ is the gradient operator, *T* is the temperature (K), *λ* is the thermal conductivity (W/mK), and Q is the total heat of the materials (J).

## 4. Results and Discussion

### 4.1. Calculation Thermal Conductivity of Composite Materials 

**Figure 3:**
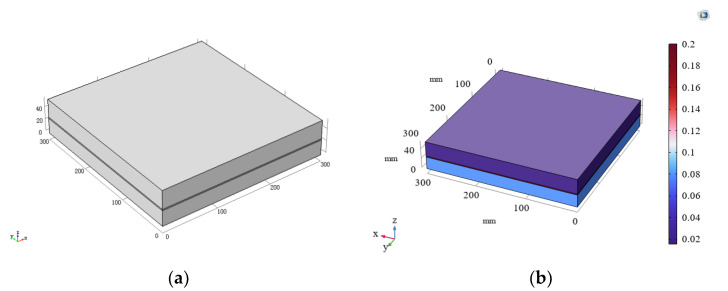
Model of composite materials produced using COMSOL. (**a**) Establishment of model for scheme 1; (**b**) average effective thermal conductivity diagram for scheme 1.

For Scheme 1, after establishing the model and inputting the material parameters, the figure displayed in Figure 3 was produced.

The calculation results are presented in Table 3. While the error between the simulation results obtained using the COMSOL software and the measured results was small, there was still some deviation. This can be attributed to two factors. Firstly, the absence of measured material density and specific heat capacity may have led to simulation errors. Secondly, the simulation software assumes that the boundary condition around the material is thermal insulation, but this was not the case with the thermal conductivity tester.

Table 4 presents the theoretical calculation results. However, the calculation formula for the thermal conductivity of the composites does not take into account the influence of material density and specific heat capacity on solid heat transfer. Additionally, the assumption that temperature is transmitted in only one direction is significantly different from actual three-dimensional heat transfer. As a result, the calculated results were lower than those obtained through experimental measurements.

### 4.2. Influence of Porosity on Thermal Conductivity

**Figure 4:**
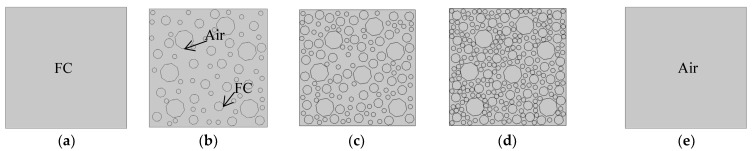
Five different porosity maps. (**a**) 0%; (**b**) 20%; (**c**) 40%; (**d**) 60%; (**e**) 100%.

In this study, the indoor environment was assumed to be 20 °C, with the hot plate (upper surface) set to 35 °C and the cold plate (lower surface) set to 15 °C. Both sides of the sample were open boundaries. The random distribution of the pore structure was established using MATLAB, and the thermal conductivity of concrete of five different porosities (0%, 20%, 40%, 60%, and 100%) was measured, as is shown in Figure 4 [49]. The initial thermal conductivity of the FC aggregate was set to the thermal conductivity of the FC, while the air inside the pores was assumed to have zero thermal conductivity.

**Figure 5:**
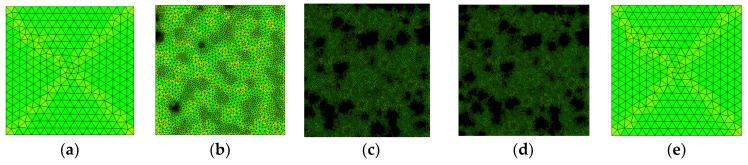
Mesh generation and independence tests of five different kinds of porosity. (**a**) 0%; (**b**) 20%; (**c**) 40%; (**d**) 60%; (**e**) 100%.

The concrete test blocks shown in Figure 5 were meshed and tested for independence, and the quality of meshing is indicated by the color (green represents better meshing). The maximum unit size was 6.7 mm, the minimum unit size was 0.03 mm, and the maximum unit growth rate was 1.3, as is illustrated in Figure 5a,e. In Figure 5b, the maximum unit size is 3.7 mm, the minimum unit size is 0.0125 mm, and the maximum unit growth rate is 1.25. In Figure 5c, the maximum unit size is 2 mm, the minimum unit size is 0.0075 mm, and the maximum unit growth rate is 1.2. Lastly, in Figure 5d, the maximum unit size is 1 mm, the minimum unit size is 0.002 mm, and the maximum unit growth rate is 1.1.

**Figure 6:**
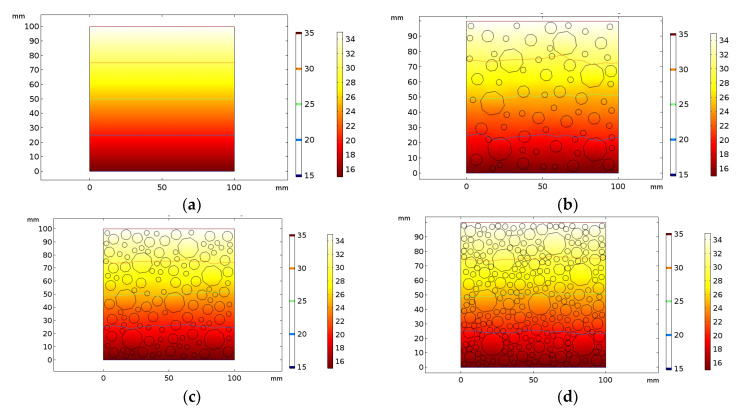
Steady-state temperatures and isotherms at different porosities. (**a**) 0%; (**b**) 20%; (**c**) 40%; (**d**) 60%.

The steady-state calculations obtained using COMSOL showed that when the porosity was 0, the temperature diffusion was uniform, and this resulted in a horizontal temperature line at the center of the concrete, as is seen in Figure 6a. As the porosity gradually increased and the number of pores increased, the temperature equilibrium curve became more twisted and complex, as is shown in Figure 6b–d. This is because, on one hand, the thermal conductivity of air is much lower than that of concrete, and this led to a thermal bridge effect at the edge of the pores. On the other hand, the presence of large pores results in unevenness in the interface, and this led to a change in the heat transfer path.

**Figure 7:**
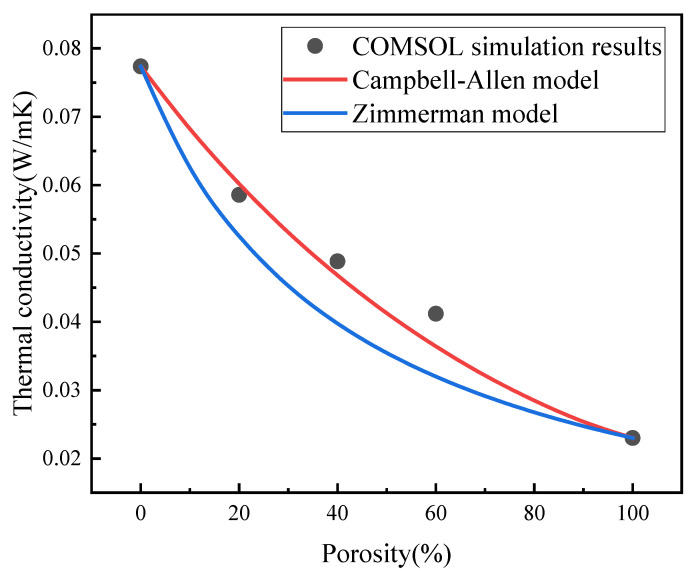
Relationship between thermal conductivity and porosity.

The calculated results were compared with two numerical theories to produce Figure 7. As is seen in Figure 7, when the porosity was 0, indicating no voids within the material, the heat conduction coefficient was equal to the thermal conductivity of the concrete. As the porosity increased, the thermal conductivity of the concrete decreased non-linearly. When the porosity reached 100%, meaning that the material consisted entirely of holes, the model became a 100 mm × 100 mm air model. Zimmerman obtained a simplified model of composite thermal conductivity using the proportions of material components. The calculation results of this model differed significantly from those of the other two methods. In the COMSOL simulation calculation, material density and specific heat capacity were considered, and the calculated thermal conductivity was in good agreement with that obtained using the Campbell-Allen model. However, Figure 7 shows that when the porosity was between 0–60%, the thermal conductivity curve calculated using COMSOL was a concave function, and this may have been a result of the slow decline in thermal conductivity which in turn was a result of the increase in the heat transfer path due to the increase in the number of holes. When the porosity increased to a certain extent (60–100%), the thermal conductivity curve became a convex function. This phenomenon may have been due to the gradual increase in the proportion of air, which has a much lower thermal conductivity than concrete, and which may have resulted in a decline in the total thermal conductivity of the composites.

## 5. Engineering Application and Design

**Figure 8:**
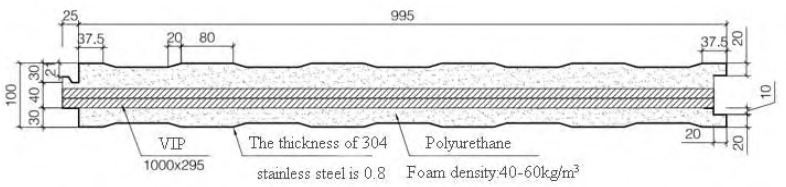
Roof panel structure design.

**Figure 9:**
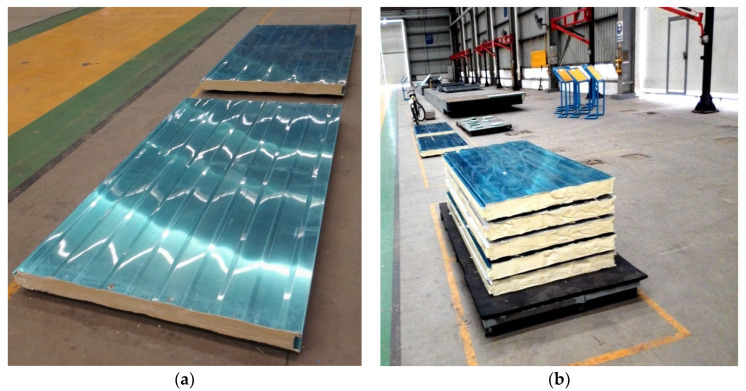
Roof panel specimens. (**a**) Main view; (**b**) side view.

This project is a part of the national key R&D plan. The roof structure design scheme and several samples were provided by Changsha Yuanda, and these are illustrated in Figure 8 and Figure 9. Specifically, two VIPs were overlapped vertically, and the remaining gaps were filled with polyurethane through cast-in situ spraying [50]. Furthermore, the fundamental properties of the raw materials used in the roof insulation system are presented in Table 5.

**Figure 10:**
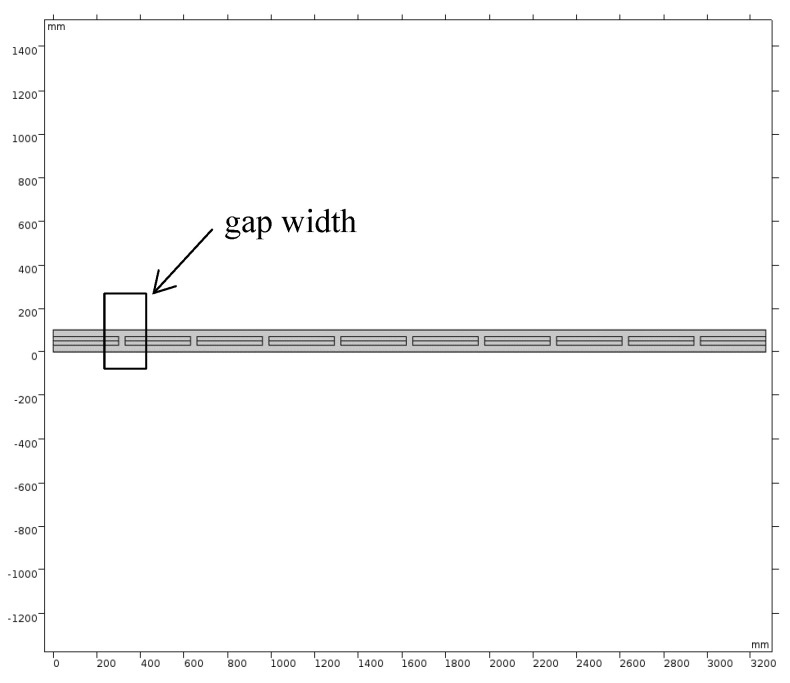
Structural model of partial product.

To facilitate cutting, a specific distance or seam width needed to be reserved between the left and right vacuum plates. This section therefore focuses on the impact of gap width on the heat transfer performance of the insulation system, which is illustrated in Figure 10.

Simulation results of slot width and heat transfer performance were obtained using the COMSOL model, and these are presented in Table 6. The relationship between gap width and heat transfer coefficient was nonlinear. When the gap width was less than 60 mm, the comprehensive heat transfer coefficient increased rapidly with the gap width. In contrast, when the gap width was greater than 60 mm, the comprehensive heat transfer coefficient decreased with the gap width. This is because the thermal conductivity of polyurethane is ten times higher than that of the vacuum plate. Hence, an increase in gap width will result in an increase in the proportion of polyurethane in the material, ultimately leading to an increase in the comprehensive heat transfer coefficient. However, when the gap width reached a certain threshold, the growth of the comprehensive heat transfer coefficient slowed down. This suggests that the proportion of polyurethane material was too high and that the thermal insulation effect of the vacuum plate can be neglected.

## 6. Conclusions

(1) By simulating the distribution of the pore structure, it is possible to qualitatively analyze the influence of pore structure and pores on the heat transfer path and thermal conductivity of insulation materials.
(2) The thermal conductivity of composite insulation materials can be obtained through theoretical calculation formulas and numerical simulation methods. When combined with experimental results, it can be seen that the thermal conductivity values calculated using the numerical simulation method are in good agreement with experimental data.
(3) In practical engineering, the gap width between materials can affect the overall heat transfer coefficient of the component. Through numerical simulation methods, the optimal gap width can be determined to be 60 mm.

## Figures and Tables

**Table 1:** Material parameters in laboratory.

Number	Material	Measured Thermal Conductivity (W/mK)	Specific Heat Capacity (J/kgK)	Density (kg/m^3^)
1	25 mm FC	0.077382	1050	247
2	30 mm RPUF	0.015302	1380	39.15
3	15 mm RPUF	0.018608	1380	39.15
4	2 mm mortar	0.2	1320	800
5	15 mm VIP	0.00669	1280	196.48
6	100 mm FC	0.077382	1050	247

**Table 2:** Experimental results of composite material schemes.

Composite Material Scheme	Measured Thermal Conductivity (W/mK)
Scheme 1	0.034557
Scheme 2	0.039583
Scheme 3	0.014944
Scheme 4	0.018104

**Table 3:** Thermal conductivity values of composite materials obtained using COMSOL.

Composite Material Scheme	Simulated Thermal Conductivity (W/mK)	Relative Error (%)
Scheme 1	0.033865	2.002
Scheme 2	0.037942	4.145
Scheme 3	0.015316	2.487
Scheme 4	0.018605	2.767

**Table 4:** Thermal conductivity of composite materials based on theoretical calculation formula.

Composite Material Scheme	Simulated Thermal Conductivity (W/mK)	Relative Error (%)
Scheme 1	0.0248517	28.08
Scheme 2	0.0368687	6.86
Scheme 3	0.010463	29.98
Scheme 4	0.016032	11.425

**Table 5:** Raw material parameters of thermal insulation system.

Materials	Thickness (mm)	Measured Thermal Conductivity (W/mK)	Specific Heat Capacity (J/kgK)	Density (kg/m^3^)
304 stainless steel	0.8	16.28	7850	500
RPUF	30	0.025	39.15	1380
VIP	20 + 20	0.0025	196.48	1280
RPUF	30	0.025	39.15	1380
304 stainless steel	0.8	16.28	7850	500

**Table 6:** Relationship between gap width and heat transfer coefficient.

Gap Width (mm)	Heat Flux (W/m^2^)	Temperature Gradient (K/m)	Thermal Conductivity (W/mK)	Heat Transfer Coefficient (W/K)
0	1.4096	240	0.005873	0.058733
10	1.6155	248.13	0.006511	0.065107
20	1.8466	256.03	0.007212	0.072124
30	2.0533	263.66	0.007788	0.077877
40	2.2494	271.12	0.008297	0.082967
50	2.4395	278.48	0.00876	0.087601
60	2.6266	285.77	0.009191	0.091913
70	2.812	293.03	0.009596	0.095963
80	2.9957	300.27	0.009977	0.099767
90	3.1798	307.48	0.010341	0.103415
100	3.363	314.69	0.010687	0.106867
110	3.5462	321.9	0.011016	0.110165
120	3.729	329.11	0.011331	0.113306
130	3.9123	336.3	0.011633	0.116334
140	4.0949	343.51	0.011921	0.119208
150	4.2777	350.72	0.012197	0.121969
160	4.4609	357.91	0.012464	0.124637
170	4.6439	365.11	0.012719	0.127192
180	4.8267	372.31	0.012964	0.129642
190	5.0094	379.51	0.0132	0.131997
200	5.1924	386.71	0.013427	0.134271

## Data Availability

Not applicable.
